# Preservation of Person-Specific Semantic Knowledge in Semantic Dementia: Does Direct Personal Experience Have a Specific Role?

**DOI:** 10.3389/fnhum.2015.00625

**Published:** 2015-11-19

**Authors:** Julie A. Péron, Pascale Piolino, Sandrine Le Moal-Boursiquot, Isabelle Biseul, Emmanuelle Leray, Laetitia Bon, Béatrice Desgranges, Francis Eustache, Serge Belliard

**Affiliations:** ^1^‘Neuroscience of Emotion and Affective Dynamics’ laboratory, Department of Psychology, University of GenevaGeneva, Switzerland; ^2^Swiss Centre for Affective Sciences, Campus Biotech, University of GenevaGeneva, Switzerland; ^3^Memory and Cognition laboratory, Institute of Psychology, University Paris Descartes, Sorbonne Paris CitéBoulogne Billancourt, France; ^4^INSERM-UMR-S894, Center of Psychiatry and Neurosciences, Sorbonne Paris Cité, University Paris DescartesParis, France; ^5^Institut Universitaire de FranceParis, France; ^6^Memory Resource and Research Centre, Department of Neurology, Rennes University HospitalRennes, France; ^7^École des Hautes Études en Santé Publique, Department of EpidemiologyRennes, France; ^8^INSERM, U1077Caen, France; ^9^UMR-S1077, Université de Caen-NormandieCaen, France; ^10^UMR-S1077, École Pratique des Hautes ÉtudesCaen, France; ^11^University Hospital, UMR-S1077Caen, France

**Keywords:** semantic memory, semantic dementia, autobiographical memory, personally familiar names, famous names, personal experience, self

## Abstract

Semantic dementia patients seem to have better knowledge of information linked to the self. More specifically, despite having severe semantic impairment, these patients show that they have more general information about the people they know personally by direct experience than they do about other individuals they know indirectly. However, the role of direct personal experience remains debated because of confounding factors such as frequency, recency of exposure, and affective relevance. We performed an exploratory study comparing the performance of five semantic dementia patients with that of 10 matched healthy controls on the recognition (familiarity judgment) and identification (biographic information recall) of personally familiar names vs. famous names. As expected, intergroup comparisons indicated a semantic breakdown in semantic dementia patients as compared with healthy controls. Moreover, unlike healthy controls, the semantic dementia patients recognized and identified personally familiar names better than they did famous names. This pattern of results suggests that direct personal experience indeed plays a specific role in the relative preservation of person-specific semantic meaning in semantic dementia. We discuss the role of direct personal experience on the preservation of semantic knowledge and the potential neurophysiological mechanisms underlying these processes.

**Abbreviations:** MMSE, Mini Mental State Examination; PIN, Person Identity Node.

## Introduction

Semantic dementia, also known as the temporal variant of frontotemporal dementia (Goulding et al., 1989; Snowden et al., 1989; Hodges et al., 1992; Hodges and Graham, 2001), is characterized by a progressive and selective disorder of semantic knowledge (Hodges et al., 1992). The selective nature of this semantic deficit, originally reported by Warrington (1975); is confirmed by good performances on day-to-day, short-term, and working memory tasks and by the preservation of visuospatial, nonverbal reasoning, phonological, and syntactic capacities (Hodges et al., 1992; Snowden et al., 1996b). This syndrome is underlain by morphological and functional alterations of a large-scale brain network, predominantly including the temporal lobes, but more specifically the temporopolar regions (anterior and inferior temporal poles). It comprises a pattern of atrophy that affects one or both hemispheres, as well as the orbitofrontal and anterior cingulate cortices, fusiform gyrus, amygdaloid complex, insula, thalamus, caudate nucleus, and the anterior part of the hippocampus (Mummery et al., 1999, 2000; Chan et al., 2001; Galton et al., 2001; Gorno-Tempini et al., 2004; Ibach et al., 2004; Short et al., 2005; Desgranges et al., 2007; La Joie et al., 2013, 2014; Viard et al., 2013, 2014).

Semantic dementia syndrome represents a model of considerable value for studying the internal organization of the semantic memory system, given that loss of meaning of concepts is not absolute (e.g., Merck et al., 2013). It also represents a unique model for studying the dynamic interplay between semantic memory and other systems, such as autobiographical memory. Indeed, one of the most striking modulatory effects of semantic memory performance in semantic dementia is related to *personal* or *autobiographical relevance*. Autobiographical information about oneself may be better preserved than impersonal (cultural or other) information. Semantic dementia patients seem to recognize their own objects, but not similar objects belonging to another person (Snowden et al., 1994, 1995; Bozeat et al., 2002; Giovannetti et al., 2006). They also recognize famous places that they have personally visited better than places they have never been to (Snowden et al., 1994; Westmacott et al., 2001). Autobiographical relevance influences the arithmetic performance of semantic dementia patients (Julien et al., 2010); similarly, they are able to categorize images or words that are relevant to their own experience but are unable to perform formal categorization tasks as well (Snowden et al., 1995). In addition, they seem to recognize and identify their relatives more easily than they do other famous individuals who are not relevant to their autobiography (Snowden et al., 1994; Graham et al., 1997; Westmacott et al., 2001, 2004; Joubert et al., 2004). This phenomenon has been called “cognitive egocentrism” (Belliard et al., 2001) in reference to the behavioral egocentrism that characterizes behavioral changes in semantic dementia (Bozeat et al., 2000; Rankin et al., 2005; Lough et al., 2006).

However, this notion is difficult to define and operationalize in the context of the performance evaluation of patients. The literature indeed provides various definitions of the notion of personal or autobiographical effect that may obscure the identification of factors that determine the preservation of semantic meaning in semantic dementia (for terminology, see **Box 1**). For some authors (Van Lancker, 1991), the concept of personal experience implies an affective or emotional relationship between the subject and an “object,” independent of the frequency of occurrence and familiarity. Other authors used the concept of personal experience in semantic dementia independently of the affective or emotional value referring to “*autobiographical significance*” (Westmacott et al., 2001, 2004), defining this notion as “*specific personal memories containing episodic, contextual details that are associated with a non-autobiographical semantic concept*” (Westmacott and Moscovitch, 2003; p. 25). Accordingly, semantic concepts that have special autobiographical significance possess a wealth of “extra” information such that their representations are colored by personal experience (Westmacott et al., 2004; p. 26). In normal subjects, this personal significance may involve various levels of autobiographical knowledge from episodic to more personal semantic representation (Piolino et al., 2007). Indeed, autobiographical memory implies different kinds of knowledge pertaining to oneself, either episodic or semantic (Piolino et al., 2009). For Snowden et al. (1994); the main critical feature of personal experience in semantic dementia is the *direct* experience between the subject and the concept, object, person, etc., rather than emotional factors or affects, personal contexts, or autobiographical significance. Only “objects” (people, locations, buildings, etc.) that have been personally and directly experienced by the subject can be included in the category of the personally relevant. For instance, Barack Obama’s face or voice may be familiar and/or frequently encountered and may even induce emotional and affective reactions. Nevertheless, as many people have never personally and/or directly met the American President, his face or voice is not personally relevant to the majority of the population. In summary, Snowden et al. (1994) suggested that personal relevance stems from the subject’s private and *direct personal experience*.

Box 1 **Concept of direct personal experience**

Although *direct personal experience* could influence the preservation of semantic knowledge in semantic dementia, the meaning of the concept is not obvious in the literature. Author’s definitions of this concept are contradictory. In fact, various terms that refer to this notion can be found in the literature, such as “*personal relevance*” (Van Lancker, 1991), “*experiential effect*” (Snowden et al., 1994, 1995; Snowden et al., 1996a; Snowden, 1999), or “*autobiographical significance*” (Westmacott et al., 2001, 2004). The literature provides three main definitions for the notion of direct personal experience.

First, according to Van Lancker (1991); the concept of direct personal experience refers to the notion of valence, which implies a relationship between the subject and an “object,” but can be distinguished from the notion of *frequency* or *familiarity*. This definition seems to be close to the notion of affective or emotional factors because “*the personally familiar objects acquire for each person an historical, unique relationship*” (Van Lancker, 1991; p. 66). In addition, Van Lancker specifies that personal relevance comes from private and cultural experience alike. Some objects or persons may be personally relevant because of the role they play in one’s private life (for instance, one’s wife or husband, house, or bunch of keys), or because of the role they play in the collective background (e.g., the Eiffel Tower or the French president, François Hollande, for the French).

Second, (Westmacott et al., 2001, 2004) and Westmacott and Moscovitch (2003); who use the term “*autobiographical significance*,” define this concept as *“specific personal memories containing episodic, contextual details that are associated with a non-autobiographical semantic concept*” (Westmacott and Moscovitch, 2003; p. 25). To illustrate their definition, the authors suggest the following example. Autobiographically significant knowledge about John Lennon may include a particular memory of hearing about his assassination. In contrast, semantic concepts include the fact that John Lennon was the singer of the Beatles or that he was married to Yoko Ono. The definition seems similar to the concept of “flashbulb memories” (Brown and Kulik, 1977), which is a memory of the personal circumstances in which one first learned of a surprising public event (Conway et al., 1994). In short, “*semantic concepts that have some special autobiographical significance possess a wealth of ‘extra’ information such that their representations are colored by personal experience”* (Westmacott and Moscovitch, 2003; p. 26). This personal experience may be of a different nature ever personal semantic or episodic (Piolino et al., 2007).

Third, Snowden et al. (1994) suggested that personal experience was not equivalent to familiarity or frequent exposure, although personally relevant concepts could also be familiar and/or frequent. Snowden et al.’s definition is very different, however, from that of Van Lancker. Indeed, their notion refers to direct and personal experience between the subject and the concept, object, person, etc., rather than to emotional factors or affects. Also in opposition to Van Lancker (1991); Snowden et al. (1994) do not consider famous persons or famous building, places, or locations to be personally relevant. Only “objects” (persons, locations, buildings, etc.) personally and directly experienced by the subject are included in the category of personally relevant. For instance, François Hollande’s face or voice might be familiar and/or frequently encountered and might even induce emotional and affective reactions. Nevertheless, many people have never personally and/or directly met the French President, making his face or voice not personally relevant for the majority of people. In contrast, faces or voices of family members, friends, or other people that the subject has directly met are considered as personally relevant to the subject and could also be considered frequent and familiar. To summarize, Snowden et al. (1994) suggest that the personal relevance notion results from the subject’s private, direct, and personal experiences.

We have adopted this latter definition because it is the only one that emphasizes a clear difference between the process for persons we have met directly and that for persons we have met indirectly or through the media. We chose to use the term *direct personal experience* to refer to this latter definition exclusively.

Snowden et al. (1994) evaluated the ability of semantic dementia patients to recognize and identify the names of personally relevant people (family members, neighbors, etc.) and those of famous people. They found that semantic dementia patients performed considerably better for the former type of items than for the latter, a pattern of findings not seen in Alzheimer’s disease patients (Snowden et al., 1995). The results have been interpreted by this group (Julien et al., 2008) in the context of the script theory originally developed by Funnell (2001). Accordingly, conceptual knowledge would be represented at different levels of abstraction, from information that is embedded in specific contexts relating to personal experience, to information that is relatively context free. In semantic dementia, the most abstracted levels of knowledge would become compromised so that patients become increasingly reliant on meaning that is grounded in personal, everyday experience (Julien et al., 2008).

That being said and by contrast, other authors (Greene and Hodges, 1996; Graham and Hodges, 1997; Hodges and Graham, 1998; Graham, 1999; Graham et al., 1999, 2000) have suggested that direct personal experience has no effect on previously established semantic memory in semantic dementia. Graham et al. (1997) investigated the hypothesis put forward by Snowden et al. (1994) by testing familiarity and identification abilities in relation to personally familiar names and famous names in two case studies of semantic dementia. Although Graham et al. (1997) found that personally familiar names were more likely to be correctly recognized as familiar than were the names of celebrities, unlike Snowden et al. (1994); they discovered that the identification of these familiar names was severely impaired. In order to explain the impact of direct personal experience on their recognition task, Graham et al. (1997) raised the possibility of methodological bias: (i) the *frequency of exposure* could be greater for personally relevant names than for celebrities’ names; (ii) “*there must be a stronger emotive quality to episodes in which one plays an active role compared to those one hears about or sees via the media”* (Hodges and Graham, 1998; p. 819); and (iii) the *recency of autobiographical experiences* could be the key factor in determining the preservation of semantic knowledge. In light of their results from the identification task, the authors concluded that the productions of the semantic dementia patients do not correspond to genuine semantic knowledge but rather to knowledge that is based on episodic memory or over-rehearsed and automatic processes.

We therefore aimed at clarifying the role of direct personal experience in the preservation of meaning in persons with semantic dementia. Accordingly, the present study was based on the construction of individual and idiosyncratic protocols, avoiding methodological confounding factors emphasized by Graham et al. (1997). Caregivers were asked to rate each name for (i) frequency of encounter; (ii) emotional relevance; and (iii) recency of exposure. The famous vs. personally familiar names were matched for frequency of exposure and for affective importance according to the caregivers’ ratings. Moreover, all selected items referred to people the participant had known for at least 10 years and to people the subject had met at least once in the year preceding the investigation. Our goal was to determine whether direct personal experience should indeed be regarded as a specific contributing factor in the ability of semantic dementia patients to recognize and identify individuals in their everyday lives. We compared the performance of five semantic dementia patients with 10 matched healthy controls on the recognition (familiarity judgment) and the identification (biographic information recall) of names of persons for whom they did or did not have direct personal experience (i.e., personally familiar names vs. names of celebrities). We expected an effect of direct personal experience on the performance of semantic dementia patients, unlike that of healthy controls. That is, we expected semantic dementia patients to show better performance when person’s names were embedded in a personal context after we controlled for bias evoked in the literature.

## Materials and Methods

### Participants

Two groups took part in the experiment: a study group consisting of semantic dementia patients and a healthy control group. This study was carried out in accordance with the recommendations of the International Committee of Medical Journal Editors (and approved by the local ethics committee, University Hospital of Rennes, France) with written informed consent from all participants. All participants gave written informed consent in accordance with the Declaration of Helsinki.

#### Semantic Dementia

We examined five semantic dementia patients (one woman and four men), with a median age at the time of the assessment of 69.6 years (range: 54–74 years). All had a history of insidiously progressive semantic disorder extending over several years (mean duration = 2.6 years, range: 1–5 years) and had been diagnosed with semantic dementia according to the criteria established by Neary and Snowden (1988) (Mini Mental State Examination (MMSE), mean = 18.8, range: 13–27; Folstein et al., 1975). At their initial presentation, all patients had reported problems in naming and comprehension. Their day-to-day memory was well preserved, and all of them could find their way around the home without getting lost. Abnormal behaviors, such as repetitive clock watching or other behavioral routines previously shown to be characteristic of semantic dementia (Snowden et al., 2001), were reported in every case. In all patients, neuroimaging (1.5-T magnetic resonance imaging) revealed atrophy of the temporal lobes. In one patient (T.A.), atrophy was located in the right lobe, in two patients (M.N. and R.J.) there was no obvious asymmetry, and in two patients (M.A. and P.G.), atrophy was located in the left lobe.

#### Healthy Controls

The healthy participants were 10 older (mean age = 68.7) healthy adults (5 men). They completed a questionnaire about their education and medical history. The inclusion of participants was based on the absence of neurological or psychiatric medical history and the absence of reports of memory problems. No medication known to impair memory was permitted. Moreover, older adults were screened for dementia by using the MMSE (> 26/30). This group was matched to the semantic dementia group for age and educational background.

### General Neuropsychological Assessment

#### Sociodemographic and Background Neuropsychological Data (Excluding Memory)

For each patient, in addition to MMSE, a general neuropsychological examination was used to explore global cognitive efficiency by means of the Mattis Dementia Rating Scale (MDRS; Mattis, 1988) and language abilities by means of irregular word reading and repetition, abstract and concrete sentence repetition, a picture-naming task, the DO80 (Deloche and Hannequin, 1997), and the token test (De Renzi and Vignolo, 1962). Visuospatial abilities were measured by the copying of a complex figure (Rey figure taken from Lezak, 1976) and by the matching of identical figures and jumbled-up figures (french test called “Protocole Montréal-Toulouse d’Evaluation des Gnosies Visuelles”, PEGV; Agniel and Joanette, 1992). Executive functions were evaluated by the modified card-sorting test (Nelson, 1976), and in addition for semantic dementia patients, by the Stroop test (Stroop, 1935) and the trail-making test, parts A and B (Reitan, 1958). Problem solving was evaluated by means of Raven’s colored progressive matrices (Raven, 1965).

#### Memory-Related Neuropsychological Data

The semantic dementia patients underwent working, episodic, and semantic memory assessments. Working memory was evaluated by means of a digit span test (forward and backward; Weschler, 1991). A visuospatial episodic learning task was conducted by using the “Test de la Ruche” (Violon and Wijns, 1984). In this test, patients had to learn the position of 10 black boxes in a 41-box matrix. Lastly, semantic memory was assessed by means of two tasks based on words and pictures: the French celebrities questionnaire (described in Piolino et al., 2003) and a semantic sorting test, which is a type of categorization task. In the latter, patients were presented with 64 words and the corresponding colored pictures. The procedure consisted of sorting the words or pictures first into superordinate categories (e.g., “animals”), and then into subordinate categories (e.g., “wild” vs. “domestic”; for a full description see Merck et al., 2013).

#### Results of the Neuropsychological Assessment

In brief, the data, reported in Table 1; clearly indicated that the semantic dementia patients displayed massive semantic memory difficulties, with anomy, surface dyslexia (all patients showed disturbance of irregular word reading), and impoverished general semantic knowledge of concepts (all patients failed the DO80 picture-naming task and the two semantic knowledge tasks). In addition, executive deficits were observed for three of the five patients. We did not notice individual differences associated with the side of atrophy. No deficit in episodic memory was observed for any of the patients.

**Table 1:** **Clinical, demographic, and neuropsychological data of each semantic dementia patient**.

	Semantic dementia patients				
	M.N	T.A.	M.A.	R.J.	P.G.
**Patient’s initials**
Age (years)	72	72	74	71	54
Duration of illness (years’ post onset)	5	3	2	1	2
MMSE	22	27	13	13	19
Educational background (years)	9	7	7	7	12
Handedness	R	R	R	R	R
Mattis (out of 144)	97	120	92	87	111
Temporal atrophy	B	R	L	B	L
**Instrumental functions**
Irregular word reading (out of 18)	**15**	**14**	**3**	**13**	**16**
Irregular word repetition (out of 18)	18	18	18	18	18
Picture naming DO80 (out of 80)	**30**	**30**	**15**	**11**	**16**
Syntactic comprehension: token test (out of 36)	33	34	26	**13**	27
Matching of identical figures PEGV (out of 10)	10	10	10	10	10
Jumbled-up figures PEGV (out of 36)	33	35	33	34	36
Copying of the Rey figure (out of 36)	36	33	36	34	35
**Executive and attentional functions**
MCST (Nelson perseverations)	**4**	**7**	2	**16**	0
Stroop (Interference score)	5	8	9	1	7
TMT B-A (s)	141	153	62	–	33
Coloured progressive matrices (out of 36)	31	27	32	26	35
**Working memory**
Direct digit span subtest of the WAISR	4	4	4	7	6
Indirect digit span subtest of the WAISR	4	4	3	4	5
**Visuospatial episodic memory**
Average of the 5 recalls^1^ (out of 10)	8.4	7.6	7.8	4.2	6.6
Recognition^1^ (out of 10)	10	10	10	**5**	10
Delayed recall^1^ (out of 10)	10	10	10	4	8
**Semantic memory**
Famous name recognition^2^	**60**	**60**	**0**	**10**	**90**
Famous face recognition^2^	**40**	**40**	**0**	**0**	**80**
Famous name identification^2^	**49**	**49**	**0**	**0**	**65**
Famous face identification^2^	**35**	**35**	**0**	**0**	**51**
Superordinate category sorting WORDS^3^	**59**	**60**	**13**	**31**	**44**
Superordinate category sorting PICTURES^3^	**64**	**64**	**64**	**60**	**63**
Subordinate functional category sorting WORDS^3^	**59**	**46**	**37**	**36**	**25**
Subordinate morphological category sorting WORDS^3^	**48**	**57**	**28**	**33**	**37**
Subordinate functional category sorting PICTURES^3^	**59**	**64**	**57**	**47**	**52**

*Bold indicates impaired performance (*p* < 0.05); dash (−) indicates test was not performed. B, bilateral; MMSE, Mini Mental State Examination; L, left; MCST, Modified Wisconsin Sorting Test; PEGV, Protocole d’Evaluation des Gnosies Visuelles (visual gnosia evaluation protocol); R, right; TMT, Trail Making Test; WAISR, Wechsler Adult Intelligence Scale Revised. ^1^Test de la Ruche; ^2^recognition and identification of celebrities; ^3^semantic sorting test*.

### Experimental Tasks

The participants were asked to recognize as familiar the names of people and then to identify those they knew either personally by direct experience or by indirect sources.

#### Material

Sixty names, divided into three categories, were used for each participant: (i) 20 personally familiar names, consisting of people the participant knew personally (family members, neighbors, or friends); (ii) 20 names involving no *direct personal experience*, consisting of famous people the participant had known before the onset of the illness; and (iii) 20 unknown names that were especially constructed for the experiment that served as distracters. The famous vs. personally familiar names were matched for the *frequency* and *recency of exposure* and for *affective importance*.

1. The personally familiar names were the names of people who were familiar or close to the participant. Names of family members, friends, or neighbors were supplied by the patients’ caregivers or participants’ family members. For each item, they were asked to date the earliest and most recent encounters between the participant and the item. All selected items referred to people the participant had known for at least 10 years and to people the subject had met at least once in the year preceding the investigation. Caregivers and family members were then asked to rate the frequency of encounters and emotional relevance between the participant and the item on two behavioral scales representing a very low to a very high value (1–10 points). All items scoring under five were regarded as low frequency or low emotional value, and all items over five were regarded as high frequency or high emotional value. Lastly, caregivers and family members were asked to provide as much semantic information as they could about each item (e.g., status, occupation, number of children, address). The aim was to get them to provide the most discriminating information possible.
2. The names of famous people who participants had no direct personal experience with were selected in consultation with each patient’s caregiver or participant’s family member. These names corresponded to celebrities (politicians, comedians, actors, TV personalities, singers, etc.) who were well-known to the participants but whom the participants had never directly and personally met. In order to select these items, we used the same procedure as for the personally familiar names.
3. The unknown names were made up of combinations of first names and surnames or of a mixture of direct personal experience surnames and fictitious first names. The caregiver or family member was asked to confirm that all the fake first name-surname associations were unknown to the participant.

After the initial selection of items (famous and personally familiar names) with each patient’s caregiver or participant’s family member, we chose the items with the highest (seven or more on Likert scales) and lowest scores (three or less on Likert scales) on the two behavioral scales in order to obtain the most discriminating items. Moreover, in the case of the personally familiar names, we excluded as many redundant items as possible (e.g., sharing identical surnames). Finally, all the items were matched in terms of consonance and country of origin (all names sounded French) and in terms of construction (all items were composed of a first name and a surname).

#### Procedure

Each participant was asked to carry out two tasks (familiarity judgment and identification recall tasks) without time constraint. The items or instructions were repeated during the tasks as many times as necessary. The total duration of the procedure was about 2 h per patient on average.

##### Familiarity judgment task

Sixty names were presented to the participants: 20 personally familiar names, 20 famous names, and 20 lures. All items were presented in both written and oral form and in random order. Prior to the experiment, the participants were informed that they would see and hear names of people in their circle of family and friends, names of celebrities, and names of people they did not know at all. For each name, the participants were asked to say whether they knew the person or not (yes-no recognition). Thereafter, the participants were asked to check their choices by placing them in their respective categories (known or unknown).

*Scoring*. The total number of correct responses by condition was calculated after the checking procedure (*maximum* = 20) and expressed in terms of percentage. The 20 lures were used to calculate the percentage of false recognitions.

##### Identification recall task

All names correctly identified as being familiar were shown to the participants again. The number of trials (questions) presented during the identification depended on the number of correct recognitions in the familiarity judgement task; thus this was different for each patient. They were then asked to provide as much semantic information as possible about each person, specifying his/her occupation, the names of his/her children, his/her address, etc. If the participants provided only vague or superordinate categorical responses (e.g., “*He is the president*” instead of “*He is the French President*), they were asked to elaborate on their answer (e.g., “*Yes, but president of which country*? *When did he become president*? *What is his political party?*”, etc). All the participants’ answers were taped on a minidisc recorder.

*Scoring*. We adopted a strict scoring system that was based on the procedure of Hodges and Graham (1998). Items classified as familiar were scored on the identification task between 0 and 2, as follows:

− 0 = “Don’t know” or an unrelated/incorrect response
− 1 = Superordinate (e.g., politician, sports personality, nephew, etc.)
− 1.5 = A definition that described the person but failed to distinguish him/her from a group of others (e.g., *John Wayne played in Westerns, Marie Dupont is my niece and Maryse’s daughter—but Maryse has several daughters*)
− 2 = A unique definition that described the person and distinguished him/her from a group of others (e.g., *Clint Eastwood starred in the film* The Good, the Bad, and the Ugly*, and also in* Dirty Harry*; Marie Dupont is my niece, she’s 22, she’s got a sister and a brother, she’s studying chemistry*)

To avoid bias, two raters performed the scoring for the identification task independently by using the anonymous interview recordings. For the personally familiar name scoring, raters used all the semantic information supplied by the caregivers or participants’ family members. The information was scored on the basis of the number of items the participant had classified as familiar according to the category (personally familiar vs. famous). For each item, the mean of both raters’ scores was calculated to obtain a maximum score of two per item and then the percentage of correct responses per category was calculated.

#### Statistical Analysis

In view of the small sample size of the two groups, non-parametric analyses were used for internal consistency. Inter-group comparisons were examined by using the Mann-Whitney *U*-test. Individual differences were examined by using Z scores. Intra-group effects were examined with Wilcoxon pairwise comparisons to evaluate the effect of the experimental condition (personally familiar names vs. famous) in each group. We also used Wilcoxon pairwise comparisons to evaluate whether “low vs. high” emotional importance and “low vs. high” frequency of encounter had an impact on performance, splitting our data set according to this variable. For each task, first, we performed intra-group comparisons, including both famous and familiar names in the same model. Second, we analyzed famous and familiar names separately. Correlation analysis was performed with Spearman’s rank test. For these analyses, the unilateral statistical level of significance was set at 0.05. The inter-rater reliability of the scoring system on the identification recall task was analyzed in a patient-by-patient fashion, using Kappa coefficients.

## Results

### Familiarity Judgment Task

**Figure 1:**
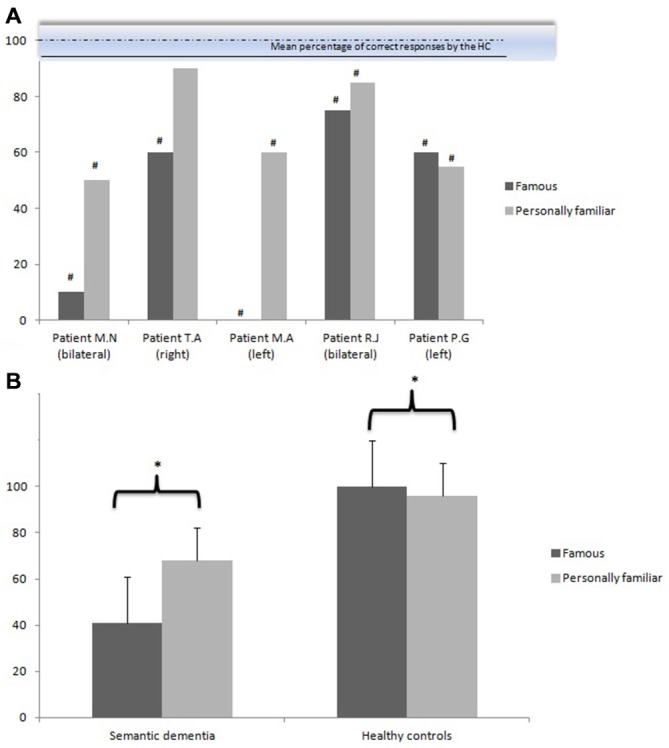
**Mean percentage of correct responses (and standard errors) on the familiarity judgment task in both experimental conditions (personally familiar names vs.famous names) by the semantic dementia group (displayed by each patient in **(A)**, at the group level in **(B)** and the healthy controls (HC) **(B)** Bilateral, right, left: laterality of the temporal atrophy**. ^#^Significant (*p* < 0.05) in comparison to the healthy controls (inter-group analyses). ^*^Significant (*p* < 0.05) in comparison to the other experimental condition (intra-group analyses).

Figure 1 illustrates the performances of the two groups on the familiarity task in both experimental conditions (personally familiar names vs. famous names).

#### Inter-Group Comparisons

Mann Whitney tests indicated that the semantic dementia group performed significantly worse than the control group for both conditions: the recognition of personally familiar (*Z* = −2.94, *p* < 0.01) and famous names (*Z* = −3.06, *p* < 0.01).

#### Intra-Group Comparisons

Analyses revealed a significant effect of experimental condition in the semantic dementia group: the personally familiar names were far better recognized as being familiar than the famous names were (*Z* = −1.75, *p* < 0.05). Moreover, analyses revealed a significant effect of experimental condition in the control group as well, but in the reverse sense: the famous names were better recognized (100%) than the personally familiar names (96%) (*Z* = 2.20, *p* < 0.05). Finally, the qualitative analysis of the lures revealed 21% false recognitions in the semantic dementia group, whereas no false recognitions were observed in the control group.

### Identification Recall Task

**Figure 2:**
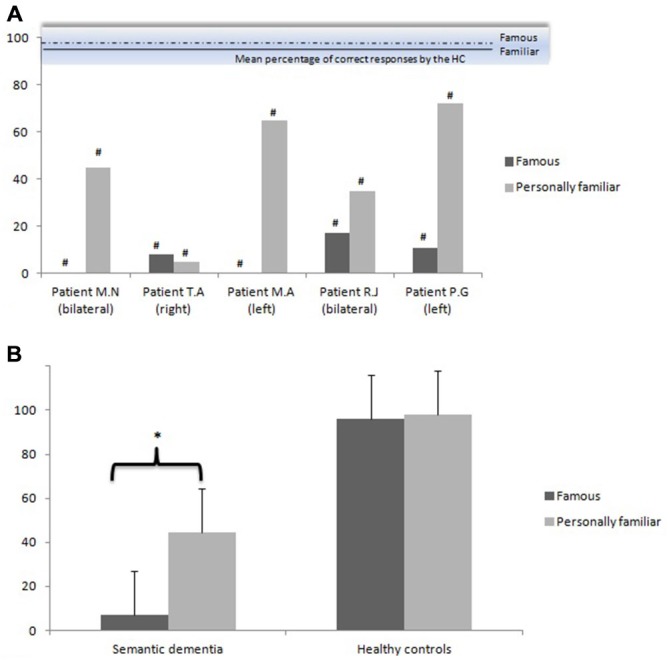
**Mean percentage of correct responses (and standard errors) on the identification free-recall task in both experimental conditions (personally familiar names vs. famous names) by the semantic dementia group (displayed by each patient in **(A)**, at the group level in **(B)** and the healthy controls (HC) **(B)** Bilateral, right, left: laterality of the temporal atrophy**. ^#^Significant (*p* < 0.05) in comparison to the healthy controls (inter-group analyses). ^*^Significant (*p* < 0.05) in comparison to the other experimental condition (intra-group analyses).

Figure 2 illustrates the performances of the two groups on the identification free-recall task in both experimental conditions (personally familiar names vs. famous names). Kappa coefficients revealed high inter-rater reliability (>0.75 for all items).

#### Inter-Group Comparisons

Similar to the familiarity judgment task, analyzes revealed that the semantic dementia group performed significantly worse than the control group for both conditions: the identification of personally familiar (*Z* = 3.06, *p* < 0.001) and famous names (*Z* = 3.06, *p* < 0.001).

#### Intra-Group Comparisons

Analyses revealed that there was a significant difference between the personally familiar names condition and the famous names condition within the semantic dementia group (*Z* = 1.75, *p* < 0.05), but no difference in the control group (*Z* = 1.01, *p* > 0.10).

Qualitatively, the pattern of responses seemed to be similar across semantic dementia patients, whether: (a) they were able to give semantic information regarding the name presented (superordinate response or even a specific response), or (b) they did not provide any response, saying that they did not know the name/person presented at all.

### Impact of “Low vs. High” Emotional Importance and Impact of “Low vs. High” Frequency of Encounter

In order to evaluate whether “low vs. high” emotional importance and “low vs. high” frequency of encounter had an impact on the performances, we split our data set according to this variable; for all comparisons, we failed to reject the null hypothesis (*p* > 0.1 for all comparisons).

### Correlations between Clinical, Demographic, and Neuropsychological Data and the Name Recognition and Identification Tasks

We found no significant correlation between the *recognition tasks* (*for both famous and familiar names*) and any of the secondary variables entered in the correlation analysis models.

However, analysis revealed a significant correlation between the *identification recall task of familiar names* and the colored progressive matrices (*r* = 0.900, *p* = 0.04), the TMT B–A (*r* = −1.000), and the number of perseverations on the MCST (*r* = −0.900, *p* = 0.04). Moreover, there was a significant correlation between the *identification recall task of famous names* and the familiarity judgment task of the famous names (*r* = 0.947, *p* = 0.01), the direct digit span subtest (*r* = 0.918, *p* = 0.03), the visuospatial episodic memory test (average of the five recalls score: *r* = 0.975, *p* = 0.005 and delayed recall score: *r* = 0.918, *p* = 0.03 of the “Test de la Ruche”), and the semantic memory test (superordinate category sorting of the pictures: *r* = 0.918, *p* = 0.03).

### Individual Differences

A measure of *Z* score was computed for each patient on familiarity and identification tasks. Table 2 shows scores for each semantic dementia patient.

*For the familiarity judgment task*, and for both experimental conditions, all the patients showed pathological scores except the only patient with right temporal atrophy (T.A.), who displayed performances in the normal range (*Z* = −1.5) on the familiarity judgment of personally familiar names. Moreover, all patients performed significantly better on the personally familiar than on the famous condition except the only patient with left temporal atrophy (P.G.).

*For the identification task*, and for both experimental conditions, all patients showed pathological scores. Moreover, and as expected, all patients performed better on the personally familiar than on the famous condition except T.A., who was the most deficient on identification of personally familiar names compared with the other patients with left or bilateral atrophy. Thus, the relative dissociation in performance between “direct personal experience” and famous condition in semantic dementia was confirmed at an individual level, with the exception of patient T.A.

**Table 2:** **Percentage of correct responses (and *Z* scores) in both experimental conditions (personally familiar names vs. famous names) in performances of each semantic dementia patient on the familiarity judgment and identification free-recall task using a strict scoring system (see Procedure)**.

	Semantic dementia patients				
Patient’s initials	M.N.	T.A.	M.A.	R.J.	P.G.
Temporal atrophy	Bilateral	Right	Left	Bilateral	Left
DPE familiarity	**50%** **(−11.5)**	90% (−1.5)	**60%** **(−9.0)**	**85%** **(−2.75)**	**55%** **(−10.25)**
Famous familiarity^*^	**10%** **(NA)**	**60%** **(NA)**	**0%** **(NA)**	**75%** **(NA)**	**60%** **(NA)**
DPE identification	**45%** **(−14.97)**	**5%** **(−26.24)**	**65%** **(−9.37)**	**35%** ** (−17.74)**	**72%** **(−9.86)**
Famous identification	**0%** **(−37.46)**	**8%** **(−34.25)**	**0%** **(−37.46)**	**17%** **(−30.67)**	**11%** ** (−33.09)**

Bold indicates impaired performance (*Z* < 1.83, *p* < 0.05; *Z* < 2.82, *p* < 0.01; *Z* < 3.25, *p* < 0.001). DPE, direct personal experience; NA, not applicable. ^*^Absence of variability across controls (correct *performance* = 100%).

## Discussion

The aim of this exploratory study was to determine whether direct personal experience should be regarded as a specific contributing factor in the relative preservation of semantic memory, especially the ability of semantic dementia patients to recognize as familiar and identify those individuals that they regularly come into contact with in their daily lives. Our results plead in favor of this hypothesis, showing that semantic dementia patients were better at recognizing personally familiar names and at retrieving knowledge about these names than about famous names. In contrast, as expected, the healthy control group showed no such effect.

The key finding of our study is the facilitation effect of direct personal experience on the recognition and identification of people’s names in semantic dementia when frequency and recency of encounter, as well as emotional relevance, were held constant across experimental conditions. Our study therefore seems to confirm the idea of earlier published studies concerning the role of direct personal experience in the relative preservation of name recognition and semantic knowledge in semantic dementia (Snowden et al., 1994; Hodges and Graham, 1998). We provided such evidence for relatively recent (within the last 10 years) personally familiar names. However, the performance of semantic dementia patients with personally familiar names did not reach the level of controls (except for the patient T.A. on recognition) and thus the direct personal experience effect seems to be a specific but moderate factor in familiarity judgment and identification (see also Julien et al., 2010).

Before we draw any inferences from our results, it is important to acknowledge several limitations of this study. First, autobiographical memory was not specifically tested in the neuropsychological assessment, which could represent a limitation, notably in order to perform correlations between this memory system and the performances on the recognition and identification of people’s names. Second, although we controlled for emotional importance and matched the two lists of names (famous vs. familiar) for this factor, we did not control for positive vs. negative valence of the stimuli. Accordingly, two different names may both have high emotional relevance, but for different reasons (i.e., an extremely positive or negative associated memory) and valence can play an important role in memory retrieval (Hofmann and Jacobs, 2014). In order to fine-grain our observations, valence should be taken into account in future studies. Third and finally, the degradation of semantic memory in semantic dementia means that the few items that remain intact could be hyperprimed (Calabria et al., 2009; Laisney et al., 2011), as in the case of familiar people in our context.

Now that our results seem to confirm the effect of direct personal experience in semantic dementia, several questions need to be addressed. First, what is the relevance (or impact) of these results on theoretical accounts of models of name recognition and identification?

Most of these models were built on the well-known and hierarchically organized cognitive model of face recognition described by Bruce and Young (1986). This model has been extended to include recognition of name and voice (e.g., Belin et al., 2004; see also Young and Bruce, 2011). It is important to keep in mind that recent findings challenged some of these propositions and alternative models are now being discussed (see Blank et al., 2014). In serial bottom-up models of name processing (Valentine et al., 1996), the presentation of a name is assumed to activate a set of name recognition units. The activation of a name recognition unit will then activate the store of semantic information about that person. This occurs through a set of multimodal nodes called person identity nodes (PINs). It is thought that familiarity judgments (i.e., recognizing whether a name is familiar) take place at the level of the PINs (Burton et al., 1990; Burton and Bruce, 1992, 1993). In addition, a single PIN has been assumed for each person. Our results suggest that direct personal experience helps to reinforce or support the PIN corresponding to that person and would have an effect on the actual semantic level (semantic system for persons), making it more resistant to the semantic dementia disease. These results are in accordance with the propositions by Snowden’s group [Julien et al. (2008) and Funnell (2001)] that, in semantic dementia, abstract semantic representations degrade, revealing representations that are more dependent on everyday personal experience.

The second question that our results raise is the following: Why does direct personal experience have a specific role in the preservation of person-specific feelings of familiarity and semantic knowledge? What is the difference between the personal experience of watching the French President on television and the personal experience of seeing/interacting with your doctor?

The two types of experiences are episodic experiences. However, one of them can be considered more distinctive and complex. Indeed, direct personal experience knowledge is usually represented more richly than is knowledge acquired by indirect personal experience through the media, in terms of sensory modality and information related to motion, space, and time. Therefore, the effect of direct personal experience may be based on multifaceted components, such as gaze direction, movements, gestures, bodily expressions and postures, and other nonverbal social information. Unlike famous people, who are media-based and perceived in two dimensions, direct experience with people concerns three dimensions and specific spatiotemporal and action contexts. In the present study, it is of interest that correlation analyses seem also to plead in favor of distinct cognitive contributions in each type of stimuli. Indeed, the identification of familiar names was significantly linked with executive functions, whereas the identification of famous names was significantly linked with memory (working, semantic, and visuospatial episodic memory systems). Moreover, some functional magnetic resonance imaging studies carried out in healthy participants have reported differential activation in the occipito-temporo-parietal junction, the precuneus, and the posterior cingulate cortex for personally familiar people as opposed to famous people (Gobbini et al., 2004; Sugiura et al., 2006). More specifically, larger responses to personally familiar names than to famous names have been found in the caudal region of the medial posterior cortex, but not in the rostral region. The temporo-parietal junction and the right posterior middle temporal gyrus have been implicated in social perception, including the processing of the actions and intentions of others (Allison et al., 2000; Castelli et al., 2000; Saxe and Kanwisher, 2003), as well as egocentric spatial judgment (Neggers et al., 2006). Taken together, these results led to the suggestion that these regions *“may play a role in egocentric visuospatial representations of objects or events in person representation*” (Sugiura et al., 2006; p. 857). Parallel to these results, imaging studies in semantic dementia converge to show a rostrocaudal gradient of alteration not only in the temporal lobe, but also in the fusiform gyrus and the cingulate cortex, with the anterior parts being more affected than the posterior parts, at least in the early stages of the disease (Chan et al., 2001; Desgranges et al., 2007; Brambati et al., 2009; Mion et al., 2010; Viard et al., 2013, 2014). Given the role of the medial posterior cortex in the recognition and identification of personally familiar names, our finding of the relative preservation of this category could be accounted for by the relative preservation of the medial posterior cortex and posterior cingulate at the beginning of the neurodegenerative process.

Finally, the issue of laterality on the preservation of personally familiar semantic meaning remains to be elucidated. In this context, the performance of T.A., who presented a right temporal atrophy, is of particular interest. Regarding the familiarity judgment task, T.A. displayed a strong effect of direct personal experience, given that his performances on the recognition of personally familiar names were *normal* in comparison to those of healthy controls (Figure 1). However, regarding the identification task, T.A.’s performance was massively impaired for personally familiar names (nearly floor performance) as well as for famous names, unlike the other patients (Figure 2). Consistent with this case report, the right lateral temporal cortex has been suggested to play a role in remote episodic retrieval (Markowitsch, 1995), ecphorizing (i.e., associating and binding retrieval cues to the retrieval itself) old episodic memories. It is thus not clear why the patients with bilateral atrophy presented a direct personal experience effect, but it is important to keep in mind that bilateral atrophy of the temporal lobe is most often predominant in the left hemisphere in the early stages of semantic dementia (Chan et al., 2001; Desgranges et al., 2007; Brambati et al., 2009; Mion et al., 2010). Further neuropsychological studies, combined with neuroimaging measures, will allow a more detailed discussion of the issue of preferential neural asymmetry of the direct personal experience effect on familiarity judgments and semantic meaning in semantic dementia. Moreover, it is also possible that the different anatomic distribution of the pathological lesions could explain the heterogeneity of the clinical individual profiles. In recent years, new disease proteins and genes have been identified in semantic dementia, leading to a better understanding of the molecular mechanisms underlying this neurodegenerative disease. The vast majority of semantic dementias are characterized by the abnormal accumulation of TDP-43, but some semantic dementia patients can also have tau pathologies: argyrophilic grain disease and corticobasal degeneration (e.g., Neumann et al., 2006; Deramecourt et al., 2010). In the speech disorder domain, the different anatomic distribution of the pathological lesions has been shown to be correlated to different language disorder profiles across patients (Deramecourt et al., 2010). It would also be interesting to characterize correlations between (person-specific) semantic disorders and pathological diagnosis. Being able to predict (ante mortem) the histological characteristics from the clinical profile is one of the major challenges of the next years.

## Conclusion

In summary, our results plead in favor of the existence of the direct personal experience factor, which may have a specific effect on the preservation of name recognition and identification in semantic dementia. That said, it appears to have only a moderate effect because recognition and identification remain deficient compared with healthy controls even in a low-demand task such as a familiarity judgment. In line with bottom-up models of name processing, the direct personal experience factor would help to support the presemantic level (PIN) and would have an effect on the semantic level (semantic system for persons). Our results now need to be confirmed and reinforced by further behavioral experiments using larger samples and exploring in more detail the nature of direct personal experience on semantic meaning through other modalities (such as faces or voices) in order to ensure that this effect is a multimodal process. Functional neuroimaging studies are also needed to explore the neural basis of boost effects of direct personal experience on the preservation of semantic memory in semantic dementia. At a more clinical level, the identification of such boost effects constitutes an appropriate target for neurorehabilitation in semantic dementia.

## Conflict of Interest Statement

The authors declare that the research was conducted in the absence of any commercial or financial relationships that could be construed as a potential conflict of interest.
